# The efficacy and safety of tislelizumab with or without tyrosine kinase inhibitor as adjuvant therapy in hepatocellular carcinoma with high-risk of recurrence after curative resection

**DOI:** 10.3389/fimmu.2025.1593153

**Published:** 2025-06-18

**Authors:** Ning Peng, Lin-Feng Mao, Jia-Yong Su, Shao-Ping Liu, Jun-Jie Ou, Shu-Chang Chen, Ze Su, Wen-Feng Li, Fu-Quan Yang, Yong-Heng Zhou, Le Li, Jian-Hong Zhong

**Affiliations:** ^1^ Hepatobiliary Surgery Department, The First Affiliated Hospital of Guangxi Medical University, Nanning, China; ^2^ Hepatobiliary Surgery Department, Guangxi Medical University Cancer Hospital, Nanning, China; ^3^ Hepatobiliary Surgery Department, Guigang City People’s Hospital, Guigang, China; ^4^ General Surgery Department, The People’s Hospital of Wuzhou, Wuzhou, China; ^5^ Hepatobiliary Pancreatic Surgery Department, The First People’s Hospital of Nanning, Nanning, China; ^6^ Hepatobiliary and Pancreatic Surgery Department, The First People’s Hospital of Yulin, Yulin, China

**Keywords:** hepatocellular carcinoma, high-risk of recurrence, tislelizumab, recurrence-free survival, adjuvant therapy

## Abstract

**Background:**

Multiple studies have demonstrated that adjuvant therapy with programmed death-1 (PD-1) inhibitors can enhance the recurrence-free survival of patients with hepatocellular carcinoma (HCC) following curative resection. This study aims to assess the efficacy and safety of adjuvant tislelizumab (a PD-1 inhibitor), with or without tyrosine kinase inhibitors, in HCC patients at high risk of recurrence.

**Methods:**

This is a retrospective, multicenter, single-arm study that enrolled patients with high-risk factors for HCC recurrence. Within 4 to 8 weeks following curative resection, participants received tislelizumab, with or without tyrosine kinase inhibitors, as adjuvant therapy until disease recurrence, unacceptable toxicity, or a maximum of 1 year. The primary endpoint was recurrence-free survival. Secondary endpoints included overall survival and adverse events.

**Results:**

Between June 2020 and January 2024, a total of 108 patients were enrolled in the study. With a median follow-up duration of 24.3 months, the 12 - month and 24 - month recurrence-free survival rates were71.3% and 59.3%, respectively. The 12 - month and 24 - month overall survival rates were 88.0% and 83.4%, respectively, and the median recurrence-free survival and median overall survival were not reached. Among these 108 patients, 43 patients (39.8%) received tislelizumab monotherapy, while 65 patients (60.2%) received tislelizumab plus tyrosine kinase inhibitors. For both groups, the median recurrence-free survival (hazard ratio 1.046, 95%CI 0.58-1.90) and median overall survival (hazard ratio 1.06, 95%CI 0.42-2.67) were not reached, with no significant difference between the two groups. Patients who received adjuvant therapy for a duration of more than 6 months had a significantly longer median recurrence-free survival compared to those who received adjuvant therapy for less than 6 months (not reached vs. 22 months, hazard ratio 2.29, 95%CI 1.14-4.61). Although there was no significant difference in overall survival between the two groups (hazard ratio 2.59, 95%CI 0.80-8.35), the overall survival tended to be higher in the group with an adjuvant therapy duration of more than 6 months. The incidence of all treatment-related adverse events and that of grade 3 or higher was 79.6% and 30.6%, respectively.

**Conclusion:**

For patients with high-risk HCC, postoperative adjuvant therapy employing tislelizumab for a duration exceeding 6 months may represent a viable strategy for reducing the risk of tumor recurrence.

## Introduction

Hepatocellular carcinoma (HCC) represents a major global health burden as the sixth most commonly diagnosed malignancy and the third leading cause of cancer-associated deaths worldwide (1). Surgical resection remains the cornerstone of curative treatment for HCC. However, the 5-year recurrence rate for patients who have undergone hepatectomy for HCC can reach up to 70%, severely compromising their long-term survival prospects (2–4). Therefore, preventing HCC recurrence is crucial for improving patients’ overall survival (OS).

Recurrence risk factors are closely associated with large tumor size, multinodular tumors, involvement of macrovascular structures or postoperative microvascular invasion, poor differentiation, and the presence of satellite lesions (5). To mitigate the recurrence rate in patients with high-risk recurrence factors, guidelines from various regions advocate for differing treatment protocols. The Chinese guideline (6) recommend adjuvant transarterial chemoembolization (TACE), hepatic artery infusion chemotherapy (HAIC), and immune checkpoint inhibitors (ICIs). The South Korean guideline (7) advocate for adjuvant immunotherapy using cytokine-induced killer cells. The USA guideline (5, 8) recommend adjuvant ICIs, whereas several current clinical guidelines from Europe (9, 10) and Japan (11) do not endorse any adjuvant therapy for the prevention of postoperative HCC recurrence. An increasing body of research indicates that the combination of ICIs and tyrosine kinase inhibitors (TKIs) has achieved remarkable therapeutic outcomes as a standard therapy for patients with unresectable advanced HCC (12–14). However, there is currently a paucity of research on its efficacy in preventing postoperative HCC recurrence, necessitating further studies to confirm its effectiveness. The IMbrave050 trial marks a significant step in the search for effective adjuvant therapies for high-risk HCC patients (15). However, this trial did not yield positive results and raises several questions regarding patient selection, endpoint robustness, and the balance between efficacy and acceptable toxicity, all of which warrant further clinical investigation. A study (16) on sintilimab monotherapy as adjuvant treatment in MVI-positive patients suggests that immunotherapy improves prognosis in high-risk recurrent populations, while also indicating the need for more refined patient selection in adjuvant therapy.

Tislelizumab, a humanized IgG4 monoclonal antibody characterized by high affinity and binding specificity for PD-1, has demonstrated preliminary antitumor activity in HCC. A randomized trial indicated that, in comparison with sorafenib, tislelizumab achieved the predetermined primary endpoint, exhibiting non-inferior OS and a 15% reduction in mortality risk (17). Consequently, it has been recommended as a first-line treatment for unresectable HCC in the Chinese clinical guideline and the NCCN guideline. However, limited studies have focused on the application of tislelizumab for preventing recurrence in HCC patients following curative resection. This multicenter retrospective analysis study explored the efficacy and safety of adjuvant tislelizumab, with or without TKIs, in preventing recurrence of HCC after curative resection.

## Methods

### Patient selection and study design

With the approval of the institutional ethics committees at each study center, a multicenter retrospective study was conducted on patients who underwent curative liver resection via laparoscopy or open surgery for HCC at six hospitals in China from June 2020 to January 2024. The inclusion criteria were as follows: (1) age 18–80 years; (2) all patients had resectable HCC tumors, good liver function (Child-Pugh: A5-B7), sufficient residual standard liver volume, and no distant metastasis in accordance with the Guidelines for the Diagnosis and Treatment of Primary Liver Cancer (2019 edition) (18); (3) all surgeries achieved R0 resection and confirmation of HCC by postoperative pathology; (4) patients with at least one high-risk factor for HCC recurrence, including tumor size >5 cm, multinodular, Edmondson-Steiner grade III–IV, macrovascular invasion or microvascular invasion, were included; (5) an Eastern Cooperative Oncology Group performance status score of 0 or 1; (6) an anticipated survival time exceeding 6 months.

The exclusion criteria were established as follows: (1) patients who did not receive ICIs or ICIs in combination with TKIs therapy following resection; (2) patients who received ICIs plus adjuvant TACE or HAIC; (3) patients who received other ICI adjuvant therapies besides tislelizumab; (4) patients who underwent neoadjuvant therapy, conversion therapy, or radiotherapy; (5) patients with a history of other malignancies; (6) patients with a history of autoimmune disease; (7) patients lacking complete case and follow-up data.

This study was carried out in compliance with the Helsinki Declaration and its subsequent revisions, and the clinical research ethics guidelines of all the research centers involved. The ethics committee waived the requirement for informed consent as the patients had consented, at the time of admission, to the analysis and publication of their anonymized medical data for research purposes.

### Adjuvant therapy protocol and duration of administration

Patients underwent reexamination within 4–8 weeks following their curative hepatectomy for a comprehensive evaluation, encompassing physical examination, hematology assessment, and radiological imaging. Patients meeting enrollment criteria were recommended by their attending physician to commence intravenous tislelizumab every 3 weeks, in accordance with the supplier’s dosage guidelines, either in combination with or without TKIs therapy, for a maximum duration of 12 months. This decision was made after consultation with their attending physician regarding the benefits and drawbacks of each treatment option. The attending physician refrained from influencing the patient’s decision in any particular direction. All patients were administered tislelizumab (200mg, intravenously, on day 1 of each 3-week cycle, BeiGene, Beijing, China), either in combination with or without TKIs, as adjuvant therapy within the time frame of 4 to 8 weeks following curative resection. The TKIs utilized included lenvatinib (Eisai, Woodcliff Lake, NJ, USA), donafenib (Suzhou Zelgen Biologics, Suzhou, China), sorafenib (Bayer, Germany), anlotinib (Nanjing Chia Tai Tianqing, Nanjing, China), and apatinib (Jiangsu Hengrui Medicine). Certain patients have experienced treatment discontinuation which attributable to economic constraints, tumor recurrence, suboptimal compliance, physician recommendations, severe treatment-related adverse events (TRAEs) or voluntary patient withdrawal. Within the scope of this study, all enrolled patients received at least one complete course of adjuvant therapy with tislelizumab, either alone or in conjunction with TKIs.

During the course of tislelizumab administration, the dosage was not arbitrarily reduced unless the treatment was discontinued, interrupted, or withheld as a result of serious TRAEs. Patients across various centers may have been administered different TKIs on a once-daily basis at dosages individualized according to body weight or as per the supplier’s specifications. In cases where serious TRAEs are attributed to the TKIs, a comprehensive and rigorous assessment must be conducted prior to appropriately reducing, interrupting, withholding, or discontinuing the TKIs. Rigorous monitoring should be maintained until the TRAEs are downgraded to grade 1 or 2 following reassessment post-medication resumption. Should a patient experience recurrent or intolerable TRAEs, adjuvant therapy should be discontinued. Immune-related adverse events were evaluated using the National Cancer Institute’s Common Terminology Criteria for Adverse Events (CTCAE), version 5.0, and immunotherapy was administered in accordance with ICI-related guidelines (19).

### Data collection

The data of enrolled patients were retrieved from medical records based on the case report form table, mainly encompassing the following aspects: (1) demographic information (age and gender); (2) medical histories (including etiology, metabolic dysfunction-associated fatty liver disease (MAFLD), alpha-fetoprotein, and liver cirrhosis); (3) clinical characteristics at diagnosis (histological type, tumor number, size of the largest tumor, macrovascular invasion, microvascular invasion, Barcelona Clinic Liver Cancer (BCLC) stage, and Child-Pugh stage); (4) Eastern Cooperative Oncology Group performance status score. MAFLD and cirrhosis were diagnosed based on postoperative pathology. The maximum diameters of the target tumors during arterial enhancement on computed tomography or magnetic resonance imaging, BCLC stage, Child-Pugh stage, tumor markers, and liver function were examined and recorded prior to postoperative adjuvant therapy. Additionally, any adverse events occurring after adjuvant therapy were also documented.

### Follow-up

Following surgery, patients underwent regular postoperative surveillance via outpatient clinic visits or telephone consultations every 2 months for 2 years, then every 6 months thereafter. The latest follow-up assessment was conducted on January 2025. The following diagnostic tests were conducted during each follow-up visit: comprehensive liver function tests, serum alpha-fetoprotein (AFP) quantification, complete hematological profiling, abdominal ultrasonography, and either contrast-enhanced magnetic resonance imaging (MRI) or computed tomography (CT) scans of the chest and abdomen. When clinically indicated, suspicious lesions were subjected to biopsy for definitive histopathological evaluation.

### Outcomes

The primary endpoint was RFS which was determined from the initial post-hepatectomy day up to the earliest occurrence of HCC recurrence or metastasis, death, or the last follow-up date. The secondary endpoint was OS which was calculated from the day immediately following hepatectomy until the date of death or the last follow-up. Other secondary endpoints included TRAEs as defined by the CTCAE.

### Statistical analysis

Continuous data were presented as mean ± standard deviation when normally distributed, or as median and interquartile range when skewed. For inter-group differences in continuous variables, significance was assessed using Student t-test if the data were normally distributed, or the Mann-Whitney U-test if the data were skewed. Categorical data were expressed as numbers and percentages. Differences in categorical variables were evaluated using Pearson’s Chi-squared test or Fisher’s exact test. RFS and OS were illustrated using Kaplan-Meier curves, and differences between groups were compared using the log-rank test. Landmark analysis was performed using R 4.4.0 software to evaluate RFS and OS who received adjuvant therapy for less than 6 months and those who received it for 6–12 months. The risk was quantified in terms of hazard ratios (HRs) and their corresponding 95% confidence intervals (CIs). Statistical data processing was performed using SPSS 25.0 software (SPSS Inc., Chicago, USA) and GraphPad Prism 7.01 software (GraphPad Software Inc., San Diego, USA). A two-tailed p-value < 0.05 was considered statistically significant for all tests.

## Results

### Patients’ characteristics

**Figure 1:**
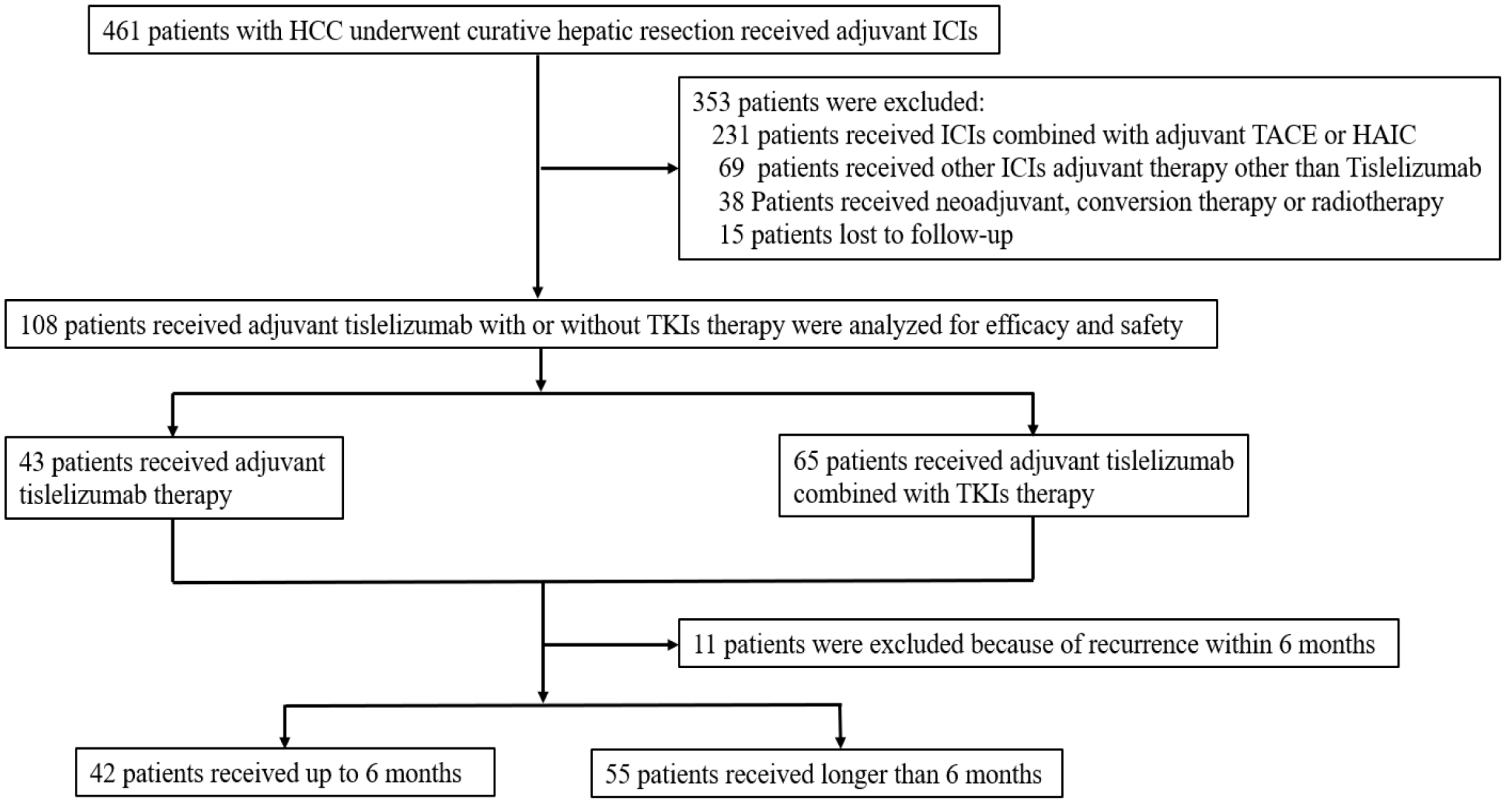
Flow diagram of patient selection: HCC, hepatocellular carcinoma; ICIs, immune checkpoint inhibitors; TKIs, tyrosine kinase inhibitors; TACE, transarterial chemoembolization; HAIC, hepatic arterial infusion chemotherapy; RFS, recurrence-free survival; OS, overall survival.

Between 10 June 2020 and 2 January 2024, 461 patients with HCC underwent curative hepatic resection and received adjuvant ICIs with or without TKIs therapy at one of six hospitals. Following exclusion criteria, 231 patients received ICIs combined with TACE or HAIC; 69 patients received non-tislelizumab ICIs; 38 patients had undergone neoadjuvant, conversion therapy or radiotherapy; and 15 patients lost to follow up were excluded. 108 patients were included for comprehensive analysis (**Figure 1**), with detailed clinical characteristics presented in **Table 1**. Among these patients, 85.2% had a chronic hepatitis B virus etiology and 72.2% presented with liver cirrhosis. Serum alpha-fetoprotein levels were elevated in 67.6% of patients. Multiple lesions (≥ 3 lesions) were found in 14.8% of patients; radiographic macrovascular invasion was detected in 22.2%, and microvascular invasion in 59.3% of patients. Tumor grades of Edmondson-Steiner III - IV were present in 51.9% of patients, and satellite lesions were found in 14.8% of patients.

**Table 1:** Baseline demographic and clinical characteristics of the 108 patients.

Variables	n=108 (%)
Age (yrs), IQR	52 (43-60)
Gender	
Male	97 (89.8)
Female	11 (10.2)
Diabetes mellitus	
Present	9 (8.3)
Absent	99 (91.7)
Fatty liver disease	
Present	21 (19.4)
Absent	87 (80.6)
Etiology	
Hepatitis B virus	92 (85.2)
Others	16 (14.8)
Liver cirrhosis	
Present	78 (72.2)
Absent	30 (27.8)
Alpha-fetoprotein, ng/ml	
>400	73 (67.6)
≤400	35 (32.4)
Number of tumors	
1	68 (63.0)
2	24 (22.2)
≥3	16 (14.8)
Tumor size (cm), mean±SD	6.5±3.6
Macrovascular invasion	
Present	24 (22.2)
Absent	84 (77.8)
Microvascular invasion	
Present	64 (59.3)
Absent	44 (40.7)
ECOG score	
0	90 (83.3)
1	18 (16.7)
Barcelona Clinic Liver Cancer stage	
A	61 (56.5)
B	18 (16.7)
C	29 (26.8)
Child-Pugh stage	
A	101 (93.5)
B	7 (6.5)
Edmondson-Steiner grade	
I-II	52 (48.1)
III-IV	56 (51.9)
Satellite lesions	
Present	16 (14.8)
Absent	92 (85.2)

Values are n (%).

IQR, interquartile range; SD, standard deviation; ECOG, Eastern Cooperative Oncology Group.

### Efficacy of adjuvant tislelizumab therapy

**Figure 2:**
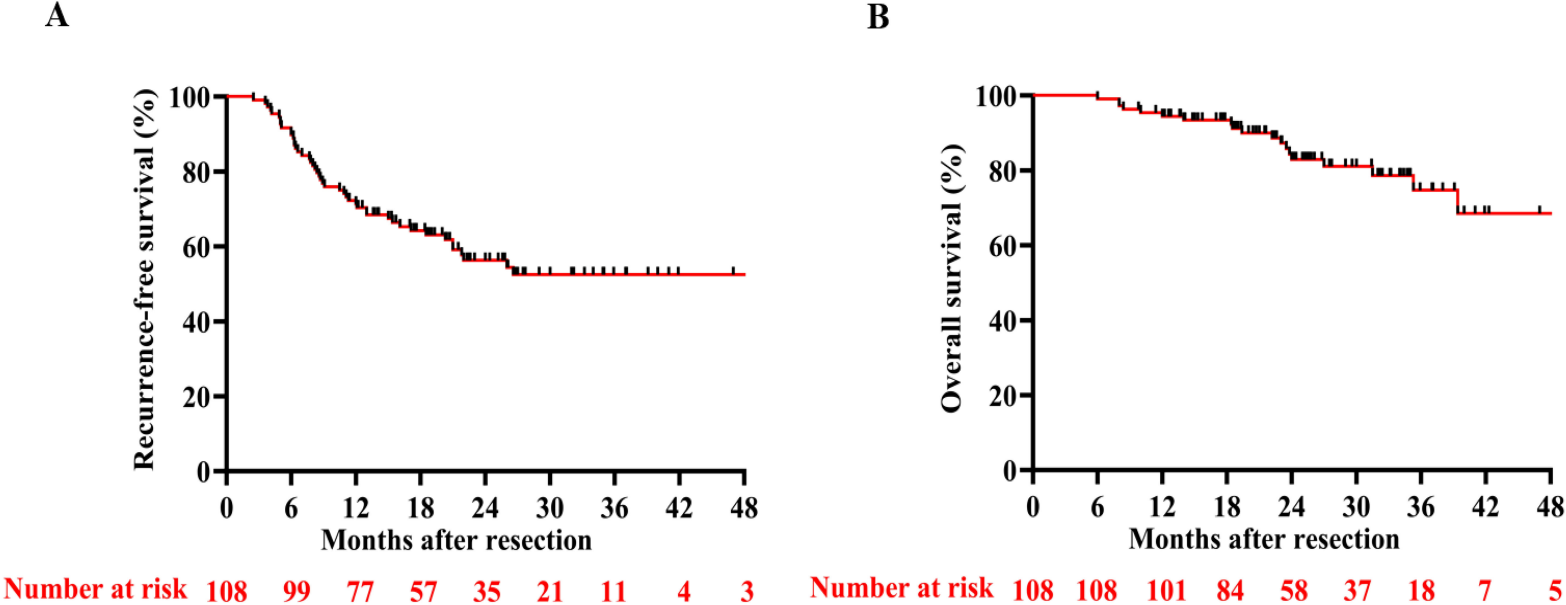
Kaplan–Meier curves of RFS **(A)** and OS **(B)** of all patients included in the study.

With a median follow-up of 24.3 months (95%CI, 23.97 - 28.05), the RFS rates at 6, 12, and 24 months were 89.8%, 71.3%, and 59.3%, respectively. The OS rates at 12 and 24 months reached 88.0% and 83.4% (**Figure 2**), respectively. By the final follow-up date, tumor recurrence had occurred in 44 patients, and 18 patients had died. Neither median RFS (**Figure 2A**) nor OS (**Figure 2B**) was reached during the observation period. Nearly all patients who experienced recurrence in the study received further single or combined treatment; the specific antitumor therapies are detailed in **Table 2**. Univariate and multivariate Cox regression analyses identified hypoalbuminemia, elevated alanine aminotransferase, and increased tumor number as independent predictive risk factors for reduced RFS (**Supplementary Table 1**). For OS, tumor size > 5 cm was the independent risk factor. (**Supplementary Table 2**).

**Table 2:** Antitumor therapies after diagnosis of hepatocellular carcinoma recurrence*.

Treatment modality	n=44 (%)
Repeat hepatic resection	7 (15.9)
Transarterial chemoembolization	29 (65.9)
Hepatic artery infusion chemotherapy	17 (38.6)
Radiofrequency or microwave ablation	17 (38.6)
Radiotherapy	4 (9.0)
Immune checkpoint inhibitor and/or tyrosine kinase inhibitor	13 (29.5)
Without antitumor therapy	2 (4.5)

Values are n (%).

*Most patients received more than one treatment.

### Comparison of RFS and OS between tislelizumab with and without TKIs group

A comparative efficacy analysis was performed to evaluate two distinct adjuvant therapy approaches: tislelizumab monotherapy versus combination therapy comprising tislelizumab plus TKIs. The study population was stratified into two treatment cohorts based on therapeutic regimen. Of the total participants, 43 patients (39.8%) received tislelizumab monotherapy, while 65 patients (60.2%) were treated with the combination of tislelizumab and TKIs. This distribution enabled a robust comparison to assess whether the incorporation of TKIs potentiates the therapeutic efficacy of tislelizumab-based adjuvant treatment. Clinicopathological variables were collected from all patients and compared using suitable statistical analysis techniques. The analysis revealed no significant differences in the aforementioned clinicopathological variables between the two groups (all *p* > 0.05; **Supplementary Table 3**).

**Figure 3:**
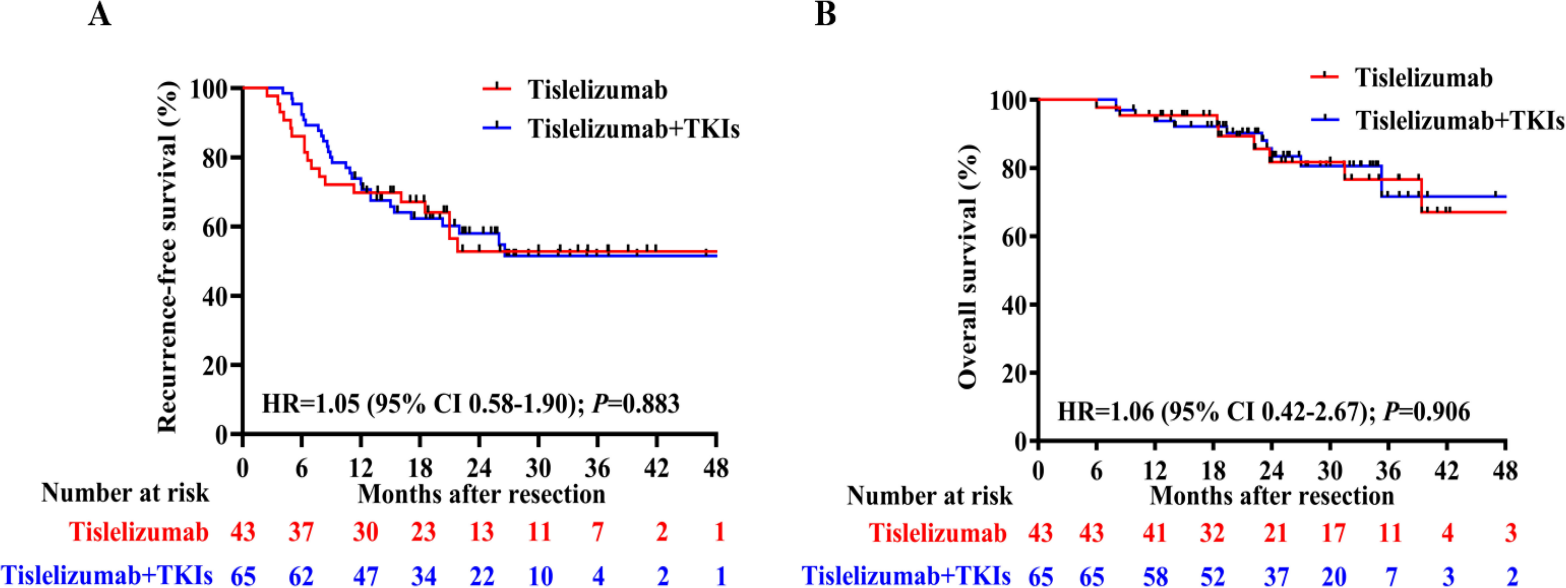
**(A)** Recurrence-free survival (RFS) and **(B)** Overall survival (OS) curves for the tislelizumab monotherapy group and combination groups.

Regarding the latest follow-up date on January 2025, 18 patients experienced tumor recurrence and 8 patients died in the tislelizumab group, while 26 patients experienced tumor recurrence and 10 patients died in the tislelizumab plus TKIs group. The median RFS between the tislelizumab group and the tislelizumab plus TKIs group was not reached, with no significant difference (HR 1.046, 95%CI 0.58–1.90, **Figure 3A**). Additionally, the median OS was not reached in either group, with no significant difference between the two groups (HR 1.06, 95%CI 0.42–2.67, **Figure 3B**).

### The impact of treatment duration on RFS and OS

**Figure 4:**
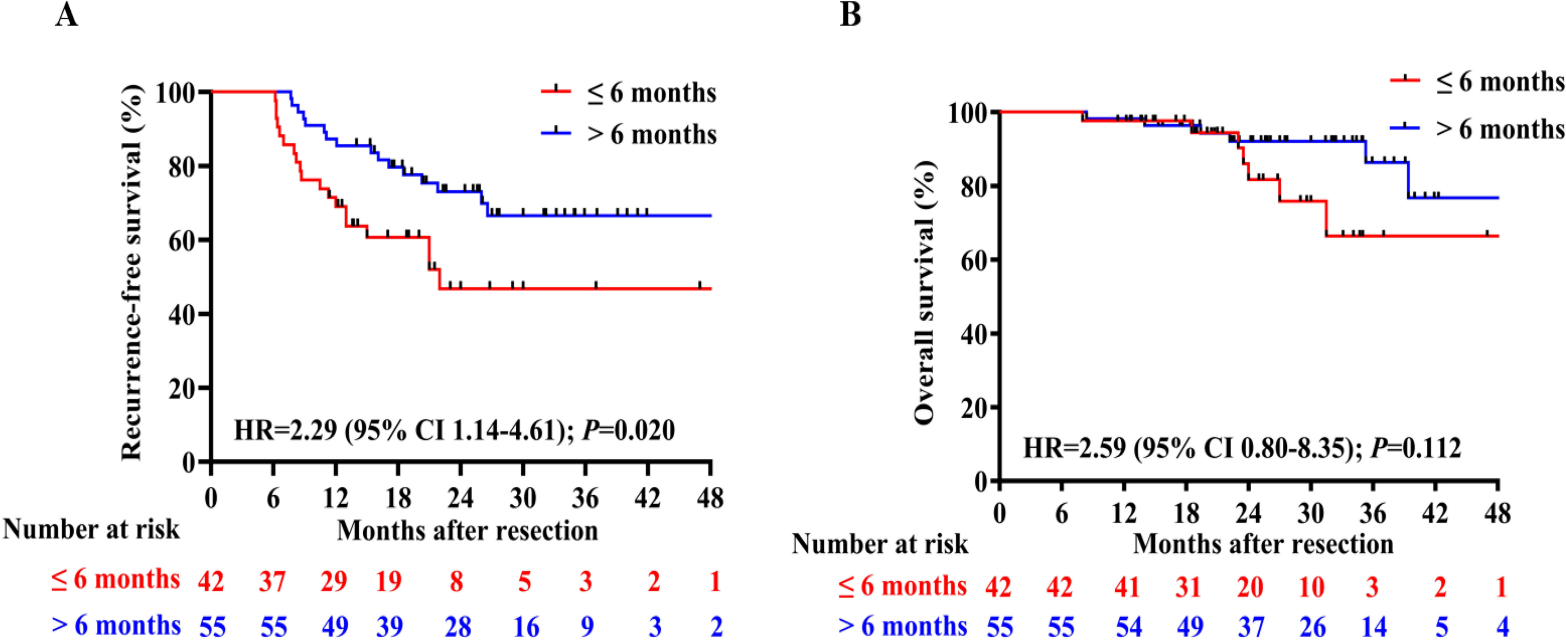
Comparison of RFS **(A)** and OS **(B)** between patients who received adjuvant therapy for up to 6 months or for more than 6 months;.

**Figure 5:**
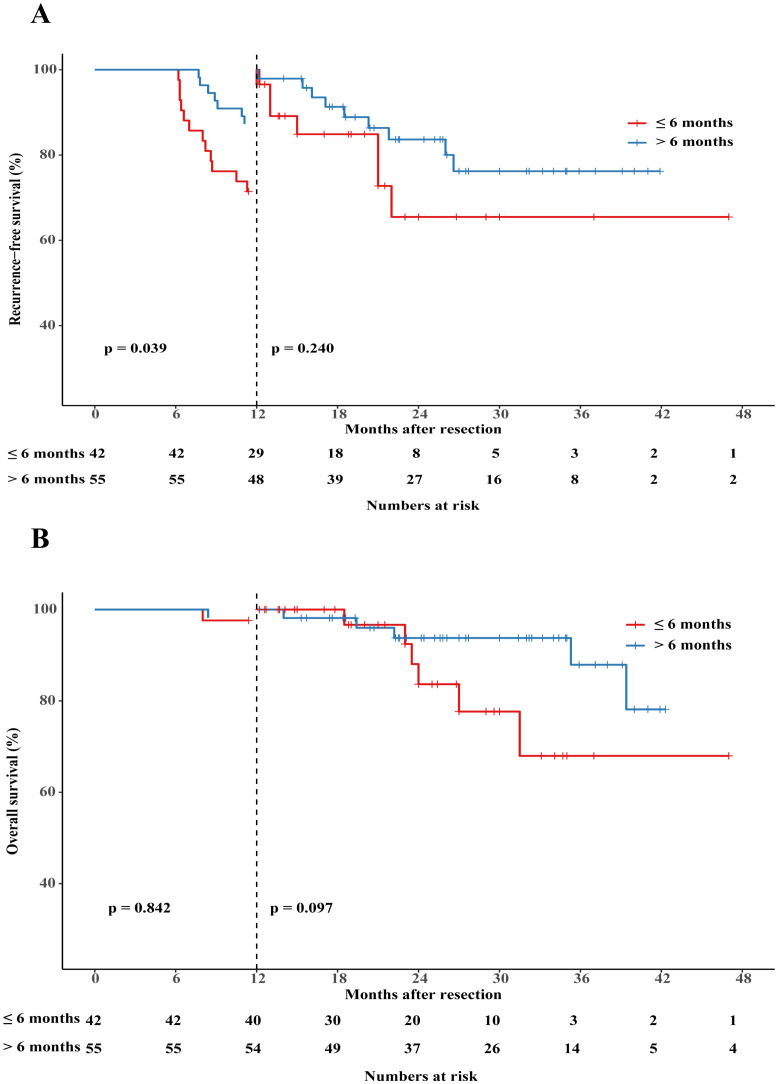
Landmark analysis discriminating between RFS **(A)** and OS **(B)** between patients who received adjuvant therapy for up to 6 months or for more than 6 months.

To assess the impact of treatment duration on patients’ RFS and OS, patients who experienced recurrence within 6 months were excluded. Subsequently, the remaining 97 patients were divided into two groups based on whether the treatment duration was longer than 6 months. The detailed clinical characteristics of patients in these two groups are presented in **Supplementary Table 4**. Patients who received adjuvant therapy for a duration of at least 6 months had a longer median RFS (p=0.020, HR 2.29, 95%CI 1.14–4.61; **Figure 4A**) and slightly higher OS (p=0.112, HR 2.59, 95% CI 0.80–8.35; **Figure 4B**) compared to those who received adjuvant therapy for less than 6 months. In order to fully control for immortal time bias, we further employed landmark analysis to evaluate the RFS and OS within and after 1 year between the two groups. The results showed that patients who received 6-12 months of adjuvant therapy exhibited a trend toward improved RFS (p=0.039) compared to those who received adjuvant therapy for less than 6 months within 1 year. However, it did not differ significantly (p=0.240) at 1–4 years’ follow-up (**Figure 5A**); Similarly, no statistically significant differences in OS were observed either within or beyond the first year of follow-up (p>0.05, **Figure 5B**).

### Safety

Adverse events occurring during treatment are highly prevalent in both immunotherapy and targeted therapy modalities. Statistical analysis reveals that the incidence of TRAEs was 65.1% in the tislelizumab monotherapy group and 89.2% in the tislelizumab plus TKIs combination group, with a statistically significant difference (p=0.003). The most frequently reported TRAEs in both groups were elevated levels of aspartate aminotransferase and alanine aminotransferase, occurring in 37.2% and 47.7% of patients, respectively. In the monotherapy cohort, the most common TRAEs included rash, hypothyroidism, and pruritus. Conversely, the combination group experienced higher frequencies of hypertension, decreased appetite, weight loss, rash, diarrhea, and abdominal pain (**Table 3**). A comparative analysis of TRAEs such as hypertension, diarrhea, joint pains or myalgia, decreased appetite, and weight loss indicated a significant disparity between the two treatment groups (p <0.05). The proportion of patients discontinuing treatment due to grade 3 or higher TRAEs was numerically lower in the tislelizumab group (18.6% vs 38.5%). Fewer patients in the tislelizumab group experienced any TRAEs leading to treatment modifications (16.3% versus 33.8%). No TRAEs were reported to cause mortality in either group.

**Table 3:** Treatment-related adverse events among patients who received adjuvant tislelizumab with or without tyrosine kinase inhibitor therapy.

Variables	Tislelizumab, n=43 (%)	Tislelizumab plus TKIs, n=65 (%)	*P*
Any	28 (65.1)	58 (89.2)	0.003
Grade ≥3	8 (18.6)	25 (38.5)	0.034
Leading to discontinuation,	2 (4.7)	6 (9.2)	0.473
Leading to treatment modification			
Any	7 (16.3)	22 (33.8)	0.049
Interrupted or withheld	7 (16.3)	17 (26.2)	0.249
Dose reduction	0 (0.0)	9 (13.8)	0.011
Incidence of TRAEs occurring in ≥10% of patients in either arm			
Hypertension	1 (2.3)	20 (30.8)	<0.001
ALT/AST increased	16 (37.2)	31 (47.7)	0.325
Blood bilirubin level increased	4 (9.3)	8 (12.3)	0.760
Fatigue	3 (7.0)	9 (13.8)	0.353
Hand-foot syndrome	1 (2.3)	7 (10.8)	0.142
Diarrhea	2 (4.7)	12 (18.5)	0.043
Pruritus	5 (11.6)	6 (9.2)	0.751
Joint pains or myalgia	0 (0.0)	9 (13.8)	0.011
Decreased appetite	2 (4.7)	16 (24.6)	0.007
Blood platelet decreased	3 (7.0)	7 (10.8)	0.737
Rash	6 (14.0)	13 (20.0)	0.453
Hypothyroidism	5 (11.6)	8 (12.3)	1.000
Weight decreased	1 (2.3)	15 (23.1)	0.002
Abdominal pain	3 (7.0)	10 (15.4)	0.237

Values are n (%).

TRAEs, Treatment-related adverse events; ALT, alanine aminotransferase; AST, aspartate aminotransferase.

## Discussion

In Chinese clinical guidelines for the management of HCC, tislelizumab is recommended as a first-line treatment for unresectable HCC. However, the evidence supporting its efficacy in preventing postoperative recurrence of HCC remains insufficient. In this study, we conducted a multicenter retrospective analysis to evaluate the efficacy and safety of tislelizumab monotherapy or in combination with TKIs in preventing postoperative recurrence of HCC. During a median follow-up period of 24.3 months, the median RFS and OS were not reached, which were significantly longer compared to patients who did not receive adjuvant therapy (the median RFS was only 16.1 months), as demonstrated in our previous study (20). This study suggests that tislelizumab alone or in combination with TKIs can significantly extend the RFS of HCC patients at high risk of recurrence after curative resection. Patients who received adjuvant therapy for at least 6 months had a significantly longer RFS than those who received it for less than 6 months. However, landmark analysis demonstrated that the between-group disparity in RFS was predominantly confined to the initial 12-month follow-up period. When compared with tislelizumab monotherapy, tislelizumab plus TKIs did not achieve a longer median RFS or overall survival, with no significant difference between the two groups. However, the combination therapy led to more grade 3 or higher TRAEs.

Although patients have undergone curative surgery or ablation treatment, the 5-year recurrence rate of HCC remains as high as 50 - 70% (21). Consequently, preventing tumor recurrence is of crucial significance for the survival prognosis of patients. With the development of TKIs and ICIs, continuous breakthroughs have been made in the treatment of unresectable HCC, achieving remarkable results (22–25). An increasing number of clinical studies are focusing on the efficacy and safety of adjuvant ICIs, with or without TKIs therapy, in preventing HCC tumor recurrence. Several studies have also demonstrated that adjuvant ICIs can improve the prognosis of patients at high risk of recurrence (16, 26–28). As a first-line treatment for unresectable HCC, there is limited research on the role of tislelizumab in preventing HCC recurrence, and more robust evidence is still urgently needed. A prospective, single-arm, phase II clinical trial (ChiCTR2200063003) aims to assess the clinical benefit of adding tislelizumab plus donafenib to TACE in patients with early-stage HCC and a high risk of recurrence. However, this study is still in progress, and no results have been obtained thus far (29). In this study, we demonstrated that adjuvant treatment with tislelizumab alone or in combination with TKIs can significantly extend the RFS of postoperative HCC patients compared to the patients in the active surveillance cohort reported in our previous studies (20, 30). This finding is consistent with previous studies (28, 31) indicating that postoperative adjuvant ICIs plus TKIs therapy can prolong the RFS of HCC patients with high-risk recurrence factors.

TKIs primarily inhibit the growth and proliferation of tumor cells and promote cell apoptosis by suppressing cell signaling transduction. They are widely used in the treatment of various cancers, significantly improving the progression-free survival and OS of patients (32, 33). Several studies have supported that the combination of ICIs with TKIs can sensitize tumors to drugs and may be superior to ICIs alone (13, 34). However, in contrast, in this study, we found that tislelizumab plus TKIs treatment did not significantly prolong patients’ RFS compared to tislelizumab monotherapy. This may be related to the fact that tislelizumab plus TKIs treatment caused more grade 3 or greater TRAEs, leading to treatment discontinuation, modification, interruption, withholding, or dose reduction during the treatment process, and may also be related to the mechanism of action of TKIs, which involves inhibiting abnormally activated tyrosine kinases in proliferating tumor cells, thereby suppressing tumor cell proliferation and angiogenesis (35). In patients who have undergone curative surgery for HCC, the body is already in a tumor-free state. Therefore, the addition of TKIs may not significantly prolong the recurrence-free survival of these patients.

Although several HCC guidelines advocate postoperative adjuvant ICI therapy for HCC patients presenting with high-risk recurrence factors, the optimal duration of such therapy remains unspecified. To date, there is no definitive answer to this question. A more precise definition of treatment duration is crucial in formulating treatment strategies for both patients and clinicians. Numerous studies recommend postoperative adjuvant therapy lasting from 6 months to 1 year in order to strike a balance between efficacy and adverse effects. Previous studies have supported the safety and efficacy of postoperative adjuvant therapy with tislelizumab for 6 months or a shorter duration of tislelizumab plus TKIs (36). Another prospective study (20) suggested that 6 months of adjuvant ICI therapy is sufficient to prolong RFS in certain patient populations. In contrast, another study posited that long-term adjuvant ICI therapy yielded enhanced efficacy for advanced non-small-cell lung cancer (37). In this investigation, we ascertained that, after excluding patients who experienced recurrence within 6 months, those receiving adjuvant therapy for a duration of at least 6 months exhibited a significantly longer RFS compared to those with less than 6 months of therapy; however, there was no significant difference in OS. The optimal duration of adjuvant therapy is contingent upon multiple factors, encompassing the patients’ specific condition, economic burden, adverse events, and so forth. Extensive and rigorous randomized controlled trials are requisite to ascertain the optimal duration of adjuvant ICI treatment.

Regarding safety, the majority of TRAEs in the tislelizumab group were grade 3 or lower, with only two patients experiencing severe complications that led to discontinuation. In contrast, the incidence of grade 3 or higher TRAEs was significantly higher in the tislelizumab plus TKIs group than that in the tislelizumab group. Therefore, we suggest that tislelizumab monotherapy may be enough in preventing postoperative recurrence in HCC patients with high-risk factors for recurrence.

This study was subject to several limitations. Firstly, being a retrospective study, it is inherently prone to biases; thus, further prospective studies are required for validation. Another primary limitation was the relatively small sample size, which may result in inadequate statistical power. Consequently, further validation through studies with larger sample sizes is warranted. Additionally, the follow-up duration in this study was relatively short, precluding the evaluation of long-term efficacy and safety. To address this, we plan to continue following up with patients to acquire extended follow-up data.

## Conclusions

In summary, these findings suggest that for patients with high-risk HCC, adjuvant therapy employing tislelizumab for a duration exceeding 6 months, either in combination with or without the use of TKIs, may represent a viable strategy for reducing the risk of tumor recurrence. Due to the cost and the TRAE of TKIs, adjuvant tislelizumab monotherapy may be the best option.

## Data Availability

The raw data supporting the conclusions of this article will be made available by the authors, without undue reservation.

## References

[1] Bray F Ferlay J Soerjomataram I Siegel RL Torre LA Jemal A. Global cancer statistics 2022: GLOBOCAN estimates of incidence and mortality worldwide for 36 cancers in 185 countries. CA Cancer J Clin. (2024) 74:229–63. doi: 10.3322/caac.21834, PMID: 38572751

[2] Zhong JH Ke Y Gong WF Xiang BD Ma L Ye XP. Hepatic resection associated with good survival for selected patients with intermediate and advanced-stage hepatocellular carcinoma. Ann Surg. (2014) 260:329–40. doi: 10.1097/SLA.0000000000000236, PMID: 24096763

[3] Zhong JH Xing BC Zhang WG Chan AW Chong CCN Serenari M. Repeat hepatic resection versus radiofrequency ablation for recurrent hepatocellular carcinoma: retrospective multicentre study. Br J Surg. (2022) 109:71–8. doi: 10.1093/bjs/znab340, PMID: 34643677

[4] Su JY Deng ZJ Teng YX Koh YX Zhang WG Zheng MH. Prognosis after hepatic resection of patients with hepatocellular carcinoma related to non-alcoholic fatty liver disease: meta-analysis. BJS Open. (2023) 7:zrac167. doi: 10.1093/bjsopen/zrac167, PMID: 36802244 PMC9939291

[5] Taddei TH Brown DB Yarchoan M Mendiratta-Lala M Llovet JM. Critical Update: AASLD Practice Guidance on prevention, diagnosis, and treatment of hepatocellular carcinoma. Hepatology. (2025). doi: 10.1097/HEP.0000000000001269, PMID: 39992051

[6] Zhou J Sun H Wang Z Cong W Zeng M Zhou W. Guidelines for the diagnosis and treatment of primary liver cancer (2022 edition). Liver Cancer. (2023) 12:405–44. doi: 10.1159/000530495, PMID: 37901768 PMC10601883

[7] Korean Liver Cancer A National Cancer Center K. 2022 KLCA-NCC Korea practice guidelines for the management of hepatocellular carcinoma. Clin Mol Hepatol. (2022) 28:583–705. doi: 10.3350/cmh.2022.0294, PMID: 36263666 PMC9597235

[8] NCCN clinical practice guidelines in Oncology (NCCN Guidelines^®^). In: *Hepatobiliary cancers* Version 2.2024.

[9] European Association for the Study of the Liver. EASL Clinical Practice Guidelines on the management of hepatocellular carcinoma. J Hepatol. (2025) 82:315–74. doi: 10.1016/j.jhep.2024.08.028, PMID: 39690085

[10] Vogel A Chan SL Dawson LA Kelley RK Llovet JM Meyer T. Hepatocellular carcinoma: ESMO Clinical Practice Guideline for diagnosis, treatment and follow-up. Ann Oncol. (2025) 36:491–506. doi: 10.1016/j.annonc.2025.02.006, PMID: 39986353

[11] Kudo M Kawamura Y Hasegawa K Tateishi R Kariyama K Shiina S. Management of hepatocellular carcinoma in Japan: JSH consensus statements and recommendations 2021 update. Liver Cancer. (2021) 10:181–223. doi: 10.1159/000514174, PMID: 34239808 PMC8237791

[12] Zhang W Tong S Hu B Wan T Tang H Zhao F. Lenvatinib plus anti-PD-1 antibodies as conversion therapy for patients with unresectable intermediate-advanced hepatocellular carcinoma: a single-arm, phase II trial. J Immunother Cancer. (2023) 11:e007366. doi: 10.1136/jitc-2023-007366, PMID: 37730273 PMC10514649

[13] Chen K Wei W Liu L Deng ZJ Li L Liang XM. Lenvatinib with or without immune checkpoint inhibitors for patients with unresectable hepatocellular carcinoma in real-world clinical practice. Cancer Immunol Immunother. (2022) 71:1063–74. doi: 10.1007/s00262-021-03060-w, PMID: 34559308 PMC10992447

[14] Deng ZJ Li L Teng YX Zhang YQ Zhang YX Liu HT. Treatments of hepatocellular carcinoma with portal vein tumor thrombus: current status and controversy. J Clin Transl Hepatol. (2022) 10:147–58. doi: 10.14218/JCTH.2021.00179, PMID: 35233384 PMC8845160

[15] Qin S Chen M Cheng AL Kaseb AO Kudo M Lee HC. Atezolizumab plus bevacizumab versus active surveillance in patients with resected or ablated high-risk hepatocellular carcinoma (IMbrave050): a randomised, open-label, multicentre, phase 3 trial. Lancet. (2023) 402:1835–47. doi: 10.1016/S0140-6736(23)01796-8, PMID: 37871608

[16] Wang K Xiang YJ Yu HM Cheng YQ Liu ZH Qin YY. Adjuvant sintilimab in resected high-risk hepatocellular carcinoma: a randomized, controlled, phase 2 trial. Nat Med. (2024) 30:708–15. doi: 10.1038/s41591-023-02786-7, PMID: 38242982

[17] Qin S Kudo M Meyer T Bai Y Guo Y Meng Z. Tislelizumab vs sorafenib as first-line treatment for unresectable hepatocellular carcinoma: A phase 3 randomized clinical trial. JAMA Oncol. (2023) 9:1651–9. doi: 10.1001/jamaoncol.2023.4003, PMID: 37796513 PMC10557031

[18] Zhou J Sun H Wang Z Cong W Wang J Zeng M. Guidelines for the diagnosis and treatment of hepatocellular carcinoma (2019 edition). Liver Cancer. (2020) 9:682–720. doi: 10.1159/000509424, PMID: 33442540 PMC7768108

[19] Schneider BJ Naidoo J Santomasso BD Lacchetti C Adkins S Anadkat M. Management of immune-related adverse events in patients treated with immune checkpoint inhibitor therapy: ASCO guideline update. J Clin Oncol. (2021) 39:4073–126. doi: 10.1200/JCO.21.01440, PMID: 34724392

[20] Li L Wu PS Liang XM Chen K Zhang GL Su QB. Adjuvant immune checkpoint inhibitors associated with higher recurrence-free survival in postoperative hepatocellular carcinoma (PREVENT): a prospective, multicentric cohort study. J Gastroenterol. (2023) 58:1043–54. doi: 10.1007/s00535-023-02018-2, PMID: 37452107

[21] Singal AG Llovet JM Yarchoan M Mehta N Heimbach JK Dawson LA. AASLD Practice Guidance on prevention, diagnosis, and treatment of hepatocellular carcinoma. Hepatology. (2023) 78:1922–65. doi: 10.1097/HEP.0000000000000466, PMID: 37199193 PMC10663390

[22] Kudo M Finn RS Qin S Han KH Ikeda K Piscaglia F. Lenvatinib versus sorafenib in first-line treatment of patients with unresectable hepatocellular carcinoma: a randomised phase 3 non-inferiority trial. Lancet. (2018) 391:1163–73. doi: 10.1016/S0140-6736(18)30207-1, PMID: 29433850

[23] Cheng AL Qin S Ikeda M Galle PR Ducreux M Kim TY. Updated efficacy and safety data from IMbrave150: Atezolizumab plus bevacizumab vs. sorafenib for unresectable hepatocellular carcinoma. J Hepatol. (2022) 76:862–73. doi: 10.1016/j.jhep.2021.11.030, PMID: 34902530

[24] Ren Z Xu J Bai Y Xu A Cang S Du C. Sintilimab plus a bevacizumab biosimilar (IBI305) versus sorafenib in unresectable hepatocellular carcinoma (ORIENT-32): a randomised, open-label, phase 2-3 study. Lancet Oncol. (2021) 22:977–90. doi: 10.1016/S1470-2045(21)00252-7, PMID: 34143971

[25] Qin S Chan SL Gu S Bai Y Ren Z Lin X. Camrelizumab plus rivoceranib versus sorafenib as first-line therapy for unresectable hepatocellular carcinoma (CARES-310): a randomised, open-label, international phase 3 study. Lancet. (2023) 402:1133–46. doi: 10.1016/S0140-6736(23)00961-3, PMID: 37499670

[26] Chen W Hu S Liu Z Sun Y Wu J Shen S. Adjuvant anti-PD-1 antibody for hepatocellular carcinoma with high recurrence risks after hepatectomy. Hepatol Int. (2023) 17:406–16. doi: 10.1007/s12072-022-10478-6, PMID: 36645648

[27] Xu X Wang MD Xu JH Fan ZQ Diao YK Chen Z. Adjuvant immunotherapy improves recurrence-free and overall survival following surgical resection for intermediate/advanced hepatocellular carcinoma a multicenter propensity matching analysis. Front Immunol. (2023) 14:1322233. doi: 10.3389/fimmu.2023.1322233, PMID: 38268916 PMC10806403

[28] Yang J Jiang S Chen Y Zhang J Deng Y. Adjuvant ICIs plus targeted therapies reduce HCC recurrence after hepatectomy in patients with high risk of recurrence. Curr Oncol. (2023) 30:1708–19. doi: 10.3390/curroncol30020132, PMID: 36826093 PMC9955678

[29] Qi W Peng W Qi X Qiu Z Wen T Li C. TIDE: adjuvant tislelizumab plus donafenib combined with transarterial chemoembolization for high-risk hepatocellular carcinoma after surgery: protocol for a prospective, single-arm, phase II trial. Front Oncol. (2023) 13:1138570. doi: 10.3389/fonc.2023.1138570, PMID: 37139154 PMC10149831

[30] Su JY Liu SP Xu XL. Treatment duration of adjuvant immune checkpoint inhibitors in hepatocellular carcinoma patients at high risk of recurrence after resection: A prospective, multicentric cohort study. Liver Cancer. (2024). doi: 10.1159/000542954 PMC12360747 40831887

[31] Li Z Han N Ren X Zhang Y Chu X. Effectiveness of TKI inhibitors combined with PD-1 in patients with postoperative early recurrence of HCC: A real-world study. Front Oncol. (2022) 12:833884. doi: 10.3389/fonc.2022.833884, PMID: 35433466 PMC9008361

[32] Dickerson H Diab A Al Musaimi O. Epidermal growth factor receptor tyrosine kinase inhibitors in cancer: current use and future prospects. Int J Mol Sci. (2024) 25:10008. doi: 10.3390/ijms251810008, PMID: 39337496 PMC11432255

[33] Atkinson TM Ryan SJ Bennett AV Stover AM Saracino RM Rogak LJ. The association between clinician-based common terminology criteria for adverse events (CTCAE) and patient-reported outcomes (PRO): a systematic review. Support Care Cancer. (2016) 24:3669–76. doi: 10.1007/s00520-016-3297-9, PMID: 27260018 PMC4919215

[34] Pinato DJ Fessas P Cortellini A Rimassa L. Combined PD-1/VEGFR blockade: A new era of treatment for hepatocellular cancer. Clin Cancer Res. (2021) 27:908–10. doi: 10.1158/1078-0432.CCR-20-4069, PMID: 33328343

[35] Du Z Lovly CM. Mechanisms of receptor tyrosine kinase activation in cancer. Mol Cancer. (2018) 17:58. doi: 10.1186/s12943-018-0782-4, PMID: 29455648 PMC5817791

[36] Pan S WS Tian J. Tislelizumab plus tyrosine kinase inhibitor versus active surveillance in patients with ablated high-risk hepatocellular carcinoma: an open-label, parallel controlled, prospective cohort study. J Clin Oncol. (2024) 24:e16238. doi: 10.1200/JCO.2024.42.16_suppl.e16238

[37] Waterhouse DM Garon EB Chandler J McCleod M Hussein M Jotte R. Continuous versus 1-year fixed-duration nivolumab in previously treated advanced non-small-cell lung cancer: checkmate 153. J Clin Oncol. (2020) 38:3863–73. doi: 10.1200/JCO.20.00131, PMID: 32910710 PMC7676888

