# Correction: The efficacy and safety of tislelizumab with or without tyrosine kinase inhibitor as adjuvant therapy in hepatocellular carcinoma with high-risk of recurrence after curative resection

**DOI:** 10.3389/fimmu.2025.1669840

**Published:** 2025-08-27

**Authors:** Ning Peng, Lin-Feng Mao, Jia-Yong Su, Shao-Ping Liu, Jun-Jie Ou, Shu-Chang Chen, Ze Su, Wen-Feng Li, Fu-Quan Yang, Yong-Heng Zhou, Le Li, Jian-Hong Zhong

**Affiliations:** ^1^ Hepatobiliary Surgery Department, The First Affiliated Hospital of Guangxi Medical University, Nanning, China; ^2^ Hepatobiliary Surgery Department, Guangxi Medical University Cancer Hospital, Nanning, China; ^3^ Hepatobiliary Surgery Department, Guigang City People’s Hospital, Guigang, China; ^4^ General Surgery Department, The People’s Hospital of Wuzhou, Wuzhou, China; ^5^ Hepatobiliary Pancreatic Surgery Department, The First People’s Hospital of Nanning, Nanning, China; ^6^ Hepatobiliary and Pancreatic Surgery Department, The First People’s Hospital of Yulin, Yulin, China

**Keywords:** hepatocellular carcinoma, high-risk of recurrence, tislelizumab, recurrence-free survival, adjuvant therapy

In the published article, there was an error in **Supplementary Tables 1** and **2**. Several units and the order in which the baseline variables appear are written incorrectly. The supplementary material has been updated in the original article.

In the published article, there was also several errors in the text. In the **Abstract**, the hazard ratio of “1.46” was erroneous due to a typographical mistake. The accurate hazard ratio value is “1.046”.

A correction has been made to **Abstract**, *Results*, paragraph 1.This sentence previously stated:

“For both groups, the median recurrence-free survival (hazard ratio 1.46, 95%CI 0.58-1.90) and median overall survival (hazard ratio 1.06, 95%CI 0.42-2.67) were not reached, with no significant difference between the two groups.”

The corrected sentence appears below:

“For both groups, the median recurrence-free survival (hazard ratio 1.046, 95%CI 0.58-1.90) and median overall survival (hazard ratio 1.06, 95%CI 0.42-2.67) were not reached, with no significant difference between the two groups.”

In the *Results*, the hazard ratio of “1.46” was erroneous due to a typographical mistake. The accurate hazard ratio value is “1.046”.

A correction has been made to **Results**, *Comparison of RFS and OS between tislelizumab with and without TKIs group*, paragraph 6. This sentence previously stated:

“The median RFS between the tislelizumab group and the tislelizumab plus TKIs group was not reached, with no significant difference (HR 1.46, 95%CI 0.58–1.90, Figure 3A).”

The corrected sentence appears below:

“The median RFS between the tislelizumab group and the tislelizumab plus TKIs group was not reached, with no significant difference (HR 1.046, 95%CI 0.58–1.90, Figure 3A).”

The original article has been updated.

