# Supplementary material for: The efficacy and safety of tislelizumab with or without tyrosine kinase inhibitor as adjuvant therapy in hepatocellular carcinoma with high-risk of recurrence after curative resection
Source: Front Immunol. 2025 Jun 18;16:1593153. doi: 10.3389/fimmu.2025.1593153 (PMC12213504; doi:10.3389/fimmu.2025.1593153)
Supplement: Supplementary file 1 [file Table1.docx]

**Supplementary Table 1.** Univariable and multivariable Cox regression analyses of factors potentially associated with recurrence-free survival.

| **Variables** | **Univariate analysis** | | **Multivariate analysis** | |  |
| --- | --- | --- | --- | --- | --- |
|  | **Hazard ratio (95%CI)** | ***p*** | **Hazard ratio (95%CI)** | ***p*** |  |
| Treatment group (with/without TKI) | | 0.96 (0.53 to 1.73) | 0.880 |  |  |
| Age (≤/>60 year) | | 0.99 (0.97 to 1.02) | 0.682 |  |  |
| Sex (male /female) | | 1.30 (0.46 to 3.61) | 0.622 |  |  |
| Diabetes mellitus (present/absent) | | 1.31 (0.52 to 3.32) | 0.569 |  |  |
| Total bilirubin, μmol/L | | 1.00 (0.99 to 1.02) | 0.807 |  |  |
| Albumin, g/L | | 0.90 (0.85 to 0.95) | <0.001 | 0.89 (0.83 to 0.95) | <0.001 |
| Alanine aminotransferase, U/L | | 1.01 (1.00 to 1.01) | <0.001 | 1.01 (1.00 to 1.01) | <0.001 |
| Alpha-fetoprotein (>/≤400 ng/mL) | | 1.36 (0.75 to 2.46) | 0.310 |  |  |
| Child-Pugh (B/A) | | 1.46 (0.52 to 4.08) | 0.473 |  |  |
| Liver cirrhosis (absent/present) | | 0.69 (0.38 to 1.27) | 0.237 |  |  |
| Fatty liver (absent/present) | | 0.59 (0.25 to 1.39) | 0.228 |  |  |
| BCLC staging | |  |  |  |  |
| A | | ref |  |  |  |
| B | | 0.77 (0.20 to 4.36) | 0.402 |  |  |
| C | | 0.77 (0.31 to 5.92) | 0.402 |  |  |
| Tumor size (<5/≥5 cm) | | 0.74 (0.41 to 1.32) | 0.307 |  |  |
| Tumor Number | |  |  |  |  |
| 1 | | ref |  |  |  |
| 2 | | 2.16 (1.46 to 6.28) | 0.016 | 2.37 (1.14 to 4.94) | 0.021 |
| ≥3 | | 2.16 (0.33 to 18.70) | 0.016 | 3.29 (0.43 to 25.0) | 0.250 |
| Macrovascular invasion (present/absent) | | 1.84 (0.98 to 3.44) | 0.059 |  |  |
| Microvascular invasion (absent/present) | | 0.62 (0.34 to 1.12) | 0.243 |  |  |
| Edmondson grade (III-IV/I-II) | | 1.36 (0.76 to 2.44) | 0.301 |  |  |
| Satellite lesions (present/absent) | | 1.79 (0.88 to 3.59) | 0.107 |  |  |

ALT: Alanine aminotransferase; AFP: Alpha-fetoprotein; BCLC: Barcelona Clinical Liver Cancer Staging; MVI: Microvascular invasion; TKI: tyrosin kinase inhibitors.
