# Supplementary material for: The efficacy and safety of tislelizumab with or without tyrosine kinase inhibitor as adjuvant therapy in hepatocellular carcinoma with high-risk of recurrence after curative resection
Source: Front Immunol. 2025 Jun 18;16:1593153. doi: 10.3389/fimmu.2025.1593153 (PMC12213504; doi:10.3389/fimmu.2025.1593153)
Supplement: Supplementary file 2 [file Table2.docx]

**Supplementary Table 2.** Univariable Cox regression analyses of factors potentially associated with overall survival.

| Variables | **Hazard ratio (95% CI)** | ***p*** |  |
| --- | --- | --- | --- |
| Treatment group (with/without TKI) | | 0.95 (0.38 to 2.38) | 0.907 |
| Age (≤/>60 year) | | 0.98 (0.94 to 1.02) | 0.387 |
| Sex (female /male) | | 0.88 (0.20 to 3.81) | 0.862 |
| Diabetes mellitus (present/absent) | | 1.21 (0.27 to 5.29) | 0.804 |
| Total bilirubin, μmol/L | | 1.01 (0.99 to 1.03) | 0.205 |
| Albumin, g/L | | 0.90 (0.85 to 0.95) | 0.563 |
| Alanine aminotransferase, U/L | | 1.00 (1.00 to 1.01) | 0.186 |
| Alpha-fetoprotein (>/≤400 ng/mL) | | 1.36 (0.55 to 3.38) | 0.511 |
| Child-Pugh (A/B) | | <0.01(0.00 to lnf) | 0.998 |
| Liver cirrhosis (absent/present) | | 0.57 (0.23 to 1.41) | 0.221 |
| Fatty liver (absent/present) | | 0.61 (0.14 to 2.65) | 0.510 |
| BCLC staging | |  |  |
| A | | ref |  |
| B | | 0.62 (0.07 to 6.65) | 0.966 |
| C | | 0.77 (0.09 to 6.16) | 0.966 |
| Tumor size (<5/≥5 cm) | | 0.33 (0.13 to 0.86) | 0.023 |
| Tumor Number | |  |  |
| 1 | | ref |  |
| 2 | | 1.80 (0.31 to 4.13) | 0.725 |
| ≥3 | | 1.80 (0.00 to Inf) | 0.725 |
| Macrovascular invasion (absent/present) | | 0.85 (0.28 to 2.58) | 0.782 |
| Microvascular invasion (absent/present) | | 0.38 (0.14 to 1.04) | 0.117 |
| Edmondson grade (III-IV/I-II) | | 1.68 (0.66 to 4.31) | 0.272 |
| Satellite lesions (present/absent) | | 2.12 (0.80 to 5.63) | 0.132 |

AFP: Alpha-fetoprotein; ALT: Alanine aminotransferase; BCLC: Barcelona Clinical Liver Cancer Staging; Inf: Infinity; MVI: Microvascular invasion; TKI: tyrosin kinase inhibitors.
