# Supplementary material for: The efficacy and safety of tislelizumab with or without tyrosine kinase inhibitor as adjuvant therapy in hepatocellular carcinoma with high-risk of recurrence after curative resection
Source: Front Immunol. 2025 Jun 18;16:1593153. doi: 10.3389/fimmu.2025.1593153 (PMC12213504; doi:10.3389/fimmu.2025.1593153)
Supplement: Supplementary file 3 [file Table3.docx]

Supplementary table 3. Comparison of clinical characteristics of patients between tislelizumab group and tislelizumab plus TKIs group.

| Variables | Tislelizumab, n=43 (%) | Tislelizumab plus TKIs, n=65 (%) | | | *p* |
| --- | --- | --- | --- | --- | --- |
| Age (yrs), IQR | 54 (49-62) | 50 (40-58) | | | 0.092 |
| Gender |  |  | | |  |
| Male | 38 (88.4) | 59 (90.8) | | | 0.751 |
| Female | 5 (11.6) | 6 (9.2) | | |  |
| Diabetes mellitus |  |  | | |  |
| Present | 2 (4.7) | 7 (10.8) | | | 0.312 |
| Absent | 41 (95.3) | 58 (89.2) | | |  |
| Fatty liver |  |  | | |  |
| Present | 11 (25.6) | 10 (15.4) | | | 0.220 |
| Absent | 32 (74.4) | 55 (84.6) | | |  |
| Etiology |  |  | | | 0.786 |
| Hepatitis B virus | 36 (83.7) | 56 (86.1) | | |  |
| Others | 7 (16.3) | | 9 (13.9) |  | |
| Liver cirrhosis |  | |  |  | |
| Present | 30 (69.8) | | 48 (73.8) | 0.666 | |
| Absent | 13 (30.2) | | 17 (26.2) |  | |
| Alpha-fetoprotein, (ng/ml) |  | |  |  | |
| >400 | 28 (65.1) | | 45 (69.2) | 0.679 | |
| ≤400 | 15 (34.9) | | 20 (30.8) |  | |
| Number of tumors |  | |  | 0.921 | |
| 1 | 27 (62.8) | | 41 (63.1) |  | |
| 2 | 9 (20.9) | | 15 (23.1) |  | |
| ≥3 | 7 (16.3) | | 9 (13.8) |  | |
| Tumor size (cm), mean±SD | 5.8±4.0 | | 7.0±3.7 | 0.110 | |
| Macrovascular invasion |  | |  |  | |
| Present | 9 (20.9) | | 15 (23.1) | 1.000 | |
| Absent | 34 (79.1) | | 50 (76.9) |  | |
| Microvascular invasion |  | |  |  | |
| Present | 25 (58.1) | | 39 (60.0) | 1.000 | |
| Absent | 18 (41.9) | | 26 (40.0) |  | |
| ECOG score |  | |  |  | |
| 0 | 36 (83.7) | | 54 (83.1) | 1.000 | |
| 1 | 7 (16.3) | | 11 (16.9) |  | |
| Barcelona Clinic Liver Cancer stage |  | |  | 0.549 | |
| A | 26 (60.5) | | 35 (53.8) |  | |
| B | 5 (11.6) | | 13 (20.0) |  | |
| C | 12 (27.9) | | 17 (26.2) |  | |
| Child-Pugh stage |  | |  |  | |
| A | 42 (97.7) | | 59 (90.8) | 0.240 | |
| B | 1 (2.3) | | 6 (9.2) |  | |
| Edmondson-Steiner grade |  | |  |  | |
| I-II | 18 (41.9) | | 34 (52.3) | 0.329 | |
| III-IV | 25 (58.1) | | 31 (47.7) |  | |
| Satellite lesions  Present  Absent | 10 (23.3)  33 (76.7) | | 6 (9.2)  59 (90.8) | 0.056 | |

Values are n (%).

TKIs, tyrosine kinase inhibitors; IQR, interquartile range; SD, standard deviation; ECOG, Eastern Cooperative Oncology Group.
