# Supplementary material for: The efficacy and safety of tislelizumab with or without tyrosine kinase inhibitor as adjuvant therapy in hepatocellular carcinoma with high-risk of recurrence after curative resection
Source: Front Immunol. 2025 Jun 18;16:1593153. doi: 10.3389/fimmu.2025.1593153 (PMC12213504; doi:10.3389/fimmu.2025.1593153)
Supplement: Supplementary file 4 [file Table4.docx]

Supplementary table 4. Clinical characteristics of patients received adjuvant treatment with different treatment duration.

| Variables | <6 months group  n=42 (%) | >6 months group n=55 (%) | *p* |
| --- | --- | --- | --- |
| Age (yrs), IQR | 50 (40.5-57) | 56 (49-62) | 0.006 |
| Gender |  |  | 1.000 |
| Male | 38 (90.5) | 49 (89.1) |  |
| Female | 4 (9.5) | 6 (10.9) |  |
| Diabetes mellitus |  |  | 0.728 |
| Present | 3 (7.1) | 6 (10.9) |  |
| Absent | 39 (92.9) | 49 (89.1) |  |
| Fatty liver |  |  | 0.804 |
| Present | 8 (19.0) | 12 (21.8) |  |
| Absent | 34 (81.0) | 43 (78.2) |  |
| Etiology |  |  | 0.257 |
| Hepatitis B virus | 38 (90.5) | 44 (80.0) |  |
| Others | 4 (9.5) | 11 (20.0) |  |
| Liver cirrhosis |  |  | 0.647 |
| Present | 32 (76.2) | 39 (70.9) |  |
| Absent | 10 (23.8) | 16 (29.1) |  |
| Alpha-fetoprotein, (ng/ml) |  |  | 0.127 |
| >400 | 32 (76.2) | 33 (60.0) |  |
| ≤400 | 10 (23.8) | 22 (40.0) |  |
| Number of tumors |  |  | 0.095 |
| 1 | 27 (64.3) | 36 (65.5) |  |
| 2 | 12 (28.6) | 8 (14.5) |  |
| ≥3 | 3 (7.1) | 11 (20.0) |  |
| Tumor size (cm), mean±SD | 6.6±3.4 | 6.4±3.6 | 0.795 |
| Macrovascular invasion |  |  | 0.614 |
| Present | 10 (23.8) | 10 (18.2) |  |
| Absent | 32 (76.2) | 45 (81.8) |  |
| Microvascular invasion |  |  | 1.000 |
| Present | 25 (59.5) | 32 (58.2) |  |
| Absent | 17 (40.5) | 23 (41.8) |  |
| ECOG score |  |  | 0.590 |
| 0 | 34 (81.0) | 47 (85.5) |  |
| 1 | 8 (19.0) | 8 (14.5) |  |
| Barcelona Clinic Liver Cancer stage | |  | 0.393 |
| A | 21 (50.0) | 35 (63.7) |  |
| B | 10 (23.8) | 7 (12.7) |  |
| C | 11 (26.2) | 13 (23.6) |  |
| Child-Pugh stage |  |  | 0.234 |
| A | 37 (88.1) | 53 (96.4) |  |
| B | 5 (11.9) | 2 (3.6) |  |
| Edmondson-Steiner grade |  |  | 0.067 |
| I-II | 25 (59.5) | 22 (40.0) |  |
| III-IV | 17 (40.5) | 33 (60.0) |  |
| Satellite lesions  Present  Absent | 6 (14.3)  36 (85.7) | 6 (10.9)  49 (89.1) | 0.758 |

Values are n (%).

IQR, interquartile range; SD, standard deviation; ECOG, Eastern Cooperative Oncology Group.
